# Supplementary material for: Why ‘elevating country voice’ is not decolonizing global health: A frame analysis of in-depth interviews
Source: PLOS Glob Public Health. 2023 Feb 23;3(2):e0001365. doi: 10.1371/journal.pgph.0001365 (PMC10022394; doi:10.1371/journal.pgph.0001365)
Supplement: S1 File — (DOCX) [file pgph.0001365.s001.docx]

| **No.** | **Topic** | **Item** |
| --- | --- | --- |
|  | **Title and abstract** |  |
| S1 | Title | Why ‘elevating country voice’ is not decolonizing global health: a frame analysis of in-depth interviews |
| S2 | Abstract | Introduction  Recent calls for global health decolonization suggest that addressing the problems of global health may require more than ‘elevating country voice’. We employed a frame analysis of the diagnostic, prognostic, and motivational framings of both discourses; and discussed the implications of convergence or divergence of these frames for global health practice and scholarship.  Methods  We used two major sources of data – review of literature and in-depth interviews with actors in global health practice and shapers of discourse around elevating country voice and decolonizing global health. Using NVivo 12, a deductive analysis approach was applied to the literature and interview transcripts with diagnostic, prognostic and motivational framings as themes.  Results  Calls for elevating country voice consider suppressed LMIC voice in global health agenda-setting and lack of country ownership of health initiatives as major problems; advancing better LMIC representation in decision making positions, and local ownership of development initiatives as solutions. The rationale for action is greater aid impact. In contrast, calls for decolonizing global health characterize coloniality, that is hierarchization of humanity and health systems, as the problem. Its prognostic framing, though still in a formative stage, includes greater acceptance of diversity in approaches to knowledge creation and health systems, and a structural transformation of global health governance. Its motivational framing is justice.  Conclusion  Conceptually and in terms of possible outcomes, the frames underlying these discourses differ. Actors’ origin and nature of involvement with global health work are markers of the frames they align with. In response to country voice elevation, operations of global health organizations in LMICs may result in greater country representation in rooms near or where power resides, but this falls short of expectations of decolonizing global health advocates. Whether governments, organizations and communities will sufficiently invest in public health to achieve decolonization remains unknown, and will determine the future of the call for decolonization and global health practice at large. |
|  | **Introduction** |  |
| S3 | Problem formulation | The call to elevate country voice in global health practice, and decolonization of global health both aim to improve global health practice, and should be mutually reinforcing. Yet, the convergence of ideas and efforts does not seem to be happening. The frame theory of Goffman (1974), Bendford and Snow (2000), and Snow et al., (2007) was reviewed. |
| S4 | Purpose or research question | How do the elevate country voice and decolonizing global health discourses frame the problem, solutions, and call to action in global health? How do these framings shape global health research and practice? |
|  | **Methods** |  |
| S5 | Qualitative approach and research paradigm | The case study qualitative approach was used. We studied key actors within the USAID [MOMENTUM suite](https://usaidmomentum.org/about/projects/) and closely adjacent partners. The USAID MOMENTUM is a suite of six maternal, newborn, child health, voluntary family planning, and reproductive health projects in 28 LMICs.  The guiding theory is the frame theory. The paradigm is interpretivist, built on Goffman’s symbolic interactionism. The rationale is the idiographic subjective knowledge which sees truth as context dependent. This paradigm is better for the study than a postpositivist approach that is largely quantitative and lacks the flexibility to address the framing of concepts and the alignment of concepts with the preferences of key actors. |
| S6 | Researcher characteristics and reflexivity | The research team is comprised of a Nigerian male academic based in Nigeria, 2 American female academics based in the US, 1 Indian female based in the UAE and 1 American male based in the US. The mix helps ensure that the pose and gaze are not ‘foreign’. The team was led by the Nigerian, a purposeful decision to address the stuck in the middle syndrome. The team is gender balanced. |
| S7 | Context | The context is global, due to the nature of the subject matter – global health research and practice. It is focused on a community of actors involved in global health practice. These actors, though largely from HICs, operate internationally, taking part in global health decision-making, agenda setting, and program implementation in LMICs. |
| S8 | Sampling strategy | The sampling strategies were purposive and snowball techniques. For in-depth interviews, we selected people occupying specific positions in MOMENTUM organizations. These positions give individuals access to information about operational policies and procedures. We stopped sampling after exhausting the list of individuals occupying such offices. We also purposefully selected individuals who are key thought leaders in the decolonization of global health debate. We selected these individuals purposefully by identifying the authors of recent publications in global health journals on this topic.  We selected published articles and grey literature purposively, focusing on publications between 2000 and 2020. New relevant publications after the original search were added. We also added a few older publications that we considered important to the discourse. The search was done on PubMed, Embase and OVID Global Health on 10/6/2020. |
| S9 | Ethical issues pertaining to human subjects | The study upheld the core principles of beneficence, respect for persons, and justice in the collection of data and management of data. We have protected the data and prevented unauthorized access to the them. We obtained ethics approval from the IRB of Johns Hopkins University, US. We obtained oral informed consent from all participants. |
| S10 | Data collection methods | In-depth interviews on Zoom between 2/2/21 and 29/4/21. The initial literature search was done on 10/6/20. |
| S11 | Data collection instruments and technologies | Open-ended semi-structured interview guide was used for the study. Interviews were audio-recorded and transcribed. |
| S12 | Units of study | Individual actors within the network constitutes the unit of analysis. 27 individuals were interviewed. 81 published articles were also reviewed. |
| S13 | Data processing | Recorded interviews were transcribed and saved in a storage drive to which only team members had access. Transcripts were numbered and anonymized. We imported the data into NVivo and did thematic coding using themes from the frame theory, that is, diagnostic framing, prognostic framing and motivational framing. All research team members read the transcripts and agreed on the coding. |
| S14 | Data analysis | We did a deductive coding, using themes from frame theory. The analysis was done by the lead author, but other team members reviewed the analysis and agreed to the coding/analysis. The interpretive approach was adopted in order to make sense of individual participant’s subjective meanings of concepts. |
| S15 | Techniques to enhance trustworthiness | All members of the team read the transcripts and reviewed the analysis to enhance the credibility of the analysis. Member checking was the approach favored by team members for convenience. |
|  | **Results/findings** |  |
| S16 | Synthesis and interpretation | Our main findings are interpretations: elevating country voice and decolonizing global health are conceptually different; the diagnostic, prognostic and motivational frames of ECV and DGH are different and one cannot be achieved by intensifying effort on the other. In a sense, they actually work at cross purposes. Our findings show how absence of frame alignment limits the actualization of decolonization of global health. |
| S17 | Links to empirical data | Analyzed documents are publicly available and are stored here: <https://drive.google.com/file/d/1HaWOE7BP0W9s3_1NMU-w7xULkhn3YdOR/view?usp=drive_web>  Interview participants were instructed that transcripts would not be shared. |
|  | **Discussion** |  |
| S18 | Integration with prior work, implications, transferability, and contributions to the field | ECV considers suppressed LMIC voice in global health agenda-setting and lack of country ownership of health initiatives as major problems; while the DGH sees the hierarchization of humanity and health systems, as the problem. This observed difference accounts for different prognostic and motivational framings. The findings agree with the frame theory position that absence of frame alignment impedes change. |
| S19 | Limitations | Limiting the study to MOMENTUM organizations, thought leaders in decolonization and documents published on the subject matter, and the small sample size reduce generalizability to other global health actors. |
|  | **Other** |  |
| S20 | Conflicts of interest | The authors do not have any conflict of interest that can influence the study. |
| S21 | Funding | This study was supported by U.S. Agency for International Development (USAID) under the terms of the Cooperative Agreement #7200AA20CA00002, led by Jhpiego and partners. The funding agency was not involved in the collection data, interpretation and reporting. |
