# Supplementary material for: Why ‘elevating country voice’ is not decolonizing global health: A frame analysis of in-depth interviews
Source: PLOS Glob Public Health. 2023 Feb 23;3(2):e0001365. doi: 10.1371/journal.pgph.0001365 (PMC10022394; doi:10.1371/journal.pgph.0001365)
Supplement: Attachment — Submitted filename: Response to reviewers.docx [file pgph.0001365.s002.docx]

Response to reviewers
Reviewer #1: The article includes an excellent review of relevant literature and seamlessly links it with key stakeholder interviews. The article is well written and contributes to our understanding of the frames of lifting country voice and decolonization. The nuanced distinction that is drawn would be of interest to global health practitioners and students.

My only suggestion/question: Is it possible to give a geographic distribution of interviewees without compromising anonymity? For example in the methods could you say that xx/28 were in the Americas, xx/28 in Asia, etc? That would present an additional level of nuance that would be of interest to readers.

The authors appreciate the comments of the reviewer. A column has been added to Table 1, on continent/region. The breakdown of the interviews appears in the text, in the second paragraph on data sources.

Reviewer #2: The authors did an extensive work and this proves the scientific relevance of the paper. As a final comment, I would suggest the authors to add in the text the definition of “country voice” as used in the document. To a reader without First hand knowledge might not be accustomed with the framework and who the "Country voice" represent.

A definition has been added, in the last two sentences in the first paragraph of the main text as suggested by the reviewer.
